# Diurnal Variation of Markers for Cholesterol Synthesis, Cholesterol Absorption, and Bile Acid Synthesis: A Systematic Review and the Bispebjerg Study of Diurnal Variations

**DOI:** 10.3390/nu11071439

**Published:** 2019-06-26

**Authors:** Maite M. Schroor, Henriette P. Sennels, Jan Fahrenkrug, Henrik L. Jørgensen, Jogchum Plat, Ronald P. Mensink

**Affiliations:** 1 Department of Nutrition and Movement Sciences, NUTRIM School of Nutrition and Translational Research in Metabolism, Maastricht University, 6200 MD Maastricht, The Netherlands; 2 Department of Clinical Biochemistry, Bispebjerg and Frederiksberg Hospital, Bispebjerg Bakke 23, DK-2400 Copenhagen NV, Denmark; 3 Department of Clinical Medicine, Faculty of Health Sciences, University of Copenhagen, Blegdamsvej 3B, DK-2200 Copenhagen N, Denmark; 4 Department of Clinical Biochemistry, Amager and Hvidovre Hospital, Kettegård Allé 30, DK-2650 Hvidovre, Denmark

**Keywords:** circadian system, diurnal rhythms, cholesterol synthesis, cholesterol absorption, bile acid synthesis

## Abstract

Human studies have shown diurnal rhythms of cholesterol and bile acid synthesis, but a better understanding of the role of the circadian system in cholesterol homeostasis is needed for the development of targeted interventions to improve metabolic health. Therefore, we performed a systematic literature search on the diurnal rhythms of cholesterol synthesis and absorption markers and of bile acid synthesis markers. We also examined the diurnal rhythms of the cholesterol synthesis markers lathosterol and desmosterol, and of the cholesterol absorption markers cholestanol, campesterol, and sitosterol in serum samples from the Bispebjerg study. These samples were collected every three hours over a 24-h period in healthy males (*n =* 24) who consumed low-fat meals. The systematic search identified sixteen papers that had examined the diurnal rhythms of the cholesterol synthesis markers lathosterol (*n =* 3), mevalonate (*n =* 9), squalene (*n =* 2), or the bile acid synthesis marker 7α-hydroxy-4-cholesten-3-one (C4) (*n =* 4). Results showed that lathosterol, mevalonate, and squalene had a diurnal rhythm with nocturnal peaks, while C4 had a diurnal rhythm with daytime peaks. Furthermore, cosinor analyses of the serum samples showed a significant diurnal rhythm for lathosterol (*cosinor p* < 0.001), but not for desmosterol, campesterol, sitosterol, and cholestanol (*cosinor p* > 0.05). In conclusion, cholesterol synthesis and bile acid synthesis have a diurnal rhythm, though no evidence for a diurnal rhythm of cholesterol absorption was found under highly standardised conditions. More work is needed to further explore the influence of external factors on the diurnal rhythms regulating cholesterol homeostasis.

## 1. Introduction

The circadian system coordinates many physiological processes in the human body around a 24-h diurnal rhythm. This system consists of a central clock located in the suprachiasmatic nucleus of the hypothalamus and multiple clocks in peripheral tissues, including the liver and intestines. To regulate these rhythms, peripheral clocks combine their own rhythms with signals from the central clock and with external factors, such as sleep and meal timing [1,2]. Evidence increasingly suggests that misalignment between the endogenous central clock and peripheral clocks negatively influences metabolic health. Circadian misalignment for example occurs when the sleep-wake cycle and the light-dark cycle are not synchronized (i.e., sleeping during the day), or when feeding rhythms and the light-dark cycle are not synchronized (i.e., eating during the night) [2]. Reviews have indeed suggested that shift workers, who regularly experience circadian misalignment, have an increased risk of developing the metabolic syndrome and cardiovascular disease [3,4]. Thereby, studies in healthy non-shift workers have shown that circadian misalignment increased blood pressure, sleeping metabolic rate, glucose, insulin, fasting plasma free fatty acids, and inflammatory marker levels, while it decreased insulin sensitivity and leptin levels [5,6,7,8].

Cholesterol homeostasis, which is regulated by the interplay between endogenous cholesterol synthesis, intestinal dietary and biliary cholesterol absorption, and bile acid synthesis and excretion, is another important determinant of metabolic health [9]. Various plasma markers reflect endogenous cholesterol synthesis (lathosterol, desmosterol, mevalonate, squalene), intestinal cholesterol absorption (sitosterol, campesterol, cholestanol), or bile acid synthesis (7α-hydroxy-4-cholesten-3-one (C4)) in both healthy and diseased people [10,11,12,13,14]. Their use as markers has been validated by analysing relations between their plasma levels and absolute measurements of endogenous cholesterol synthesis, intestinal cholesterol absorption, or bile acid synthesis [10,11,12,13,14]. It is known that cholesterol synthesis and absorption are reciprocally related. To illustrate, increased dietary cholesterol intake has been shown to increase cholesterol absorption, which was compensated for by a decreased endogenous cholesterol synthesis [15]. Treatment with ezetimibe and consumption of plant sterols and stanols has been shown to decrease cholesterol absorption, which was compensated for by an increased cholesterol synthesis [16,17]. However, large inter-individual variation in this reciprocal relation exists and people can be classified as cholesterol synthesizers (elevated cholesterol synthesis), cholesterol absorbers (elevated cholesterol absorption), or intermediate [15,16,18].

Although previous studies have demonstrated a diurnal rhythm of cholesterol synthesis and bile acid synthesis [19,20], a better understanding of the influence of the circadian system on cholesterol homeostasis is needed for targeted interventions. Knowledge of the diurnal regulation of cholesterol and bile acid metabolism may help determining when people should use specific dietary components and drugs to optimize treatment and maintain in circadian alignment. Therefore, this paper had two primary aims: 1. To give a systematic overview of the diurnal rhythms of markers reflecting endogenous cholesterol synthesis, intestinal cholesterol absorption, and bile acid synthesis, and 2. To examine the diurnal rhythms of cholesterol synthesis markers (lathosterol and desmosterol) and cholesterol absorption markers (cholestanol, campesterol, and sitosterol) in serum samples from the Bispebjerg study [21]. This study was specifically designed to examine in healthy males diurnal rhythms of several metabolic and physiological parameters, such as LDL-cholesterol, triglycerides, and glucose.

## 2. Materials and Methods

### 2.1. Systematic Review

#### 2.1.1. Search Methods

The systematic review was carried out using the Preferred Reporting Items for Systematic Review and Meta-Analyses (PRISMA) checklist [22]. The search was conducted to identify original research papers examining the diurnal rhythms of markers reflecting endogenous cholesterol synthesis, intestinal cholesterol absorption, or bile acid synthesis in humans. Potentially relevant studies were retrieved by searching the following databases: Medline, Embase, and the Cochrane Library. Databases were searched from inception until December 2018. The Medical Subject Heading (MeSH) terms used were: ((plant sterol OR plant sterols OR phytosterol OR phytosterols OR sitosterol OR campesterol OR brassicasterol OR stigmasterol OR avenasterol OR lathosterol OR desmosterol OR cholestanol OR lanosterol OR squalene OR mevalonic acid OR mevalonate OR cholesterol precursors OR cholic acid OR chenodeoxycholic acid OR deoxycholic acid OR ursodeoxycholic acid OR lithocholic acid OR C4 OR 7alphahydroxy4cholesten3one OR bile acid synthesis) AND (diurnal rhythm OR circadian rhythm) AND (humans OR human)).

#### 2.1.2. Selection Procedure

Criteria to be included were: (1) scientific papers (conference papers and posters were excluded); (2) original studies (reviews and studies based on previously published data were excluded); (3) human studies; (4) diurnal rhythms measured for ≥ 24 h; (5) levels of validated markers of cholesterol synthesis, cholesterol absorption or bile acid synthesis measured; (6) markers measured in plasma or serum (e.g., markers measured in urine were excluded); (7) at least five samples collected over a 24-h period; and (8) studies written in English. No specific publication period was considered.

The selection procedure consisted of two rounds. In the first round, two researchers (M.M.S. and R.P.M.) screened all the titles and abstracts of the papers obtained from the databases. Potentially relevant articles were selected. In the second round, the full-texts of all selected papers were read and papers that met the inclusion criteria were included. Papers that were identified through the reference lists of the selected articles were included as well. Any discrepancies between the researchers were resolved by discussion.

#### 2.1.3. Data Extraction

For each included study, the following data were extracted into an Excel spreadsheet: study information (reference number, first author, publication year), study characteristics (study design, subgroups, study setting, study duration, time between measurements), participant characteristics (*n*, age, sex, BMI, health status, medication use), marker collection (type of marker, analytical method, measurement in serum or plasma), dietary information (type of meals, time of consumption), sleep information (sleeping hours and conditions), and the main findings (mean marker concentrations at the measured time points, times of peak and nadir). In case a study presented the diurnal rhythms for each subject separately, the mean marker concentration at each time point was calculated. If mean concentrations were not provided in a table, these were estimated from graphs using a pixel ruler. Both absolute and cholesterol-standardised data were collected. In the end, only cholesterol-standardised markers were used, because these have been validated to measure cholesterol absorption, cholesterol synthesis or bile acid synthesis. Mevalonate, however, is a water-soluble compound, and was not standardised for total cholesterol.

The marker levels measured during the first 24 h of each study were presented in graphs. First, the 24-h period was divided into the following time blocks: 06:30–09:00 h; 09:30–12:00 h; 12:30–15:00 h; 15:30–18:00 h; 18:30–21:00 h; 21:30–00:00 h; 00:30–03:00 h; 03:30–06:00 h; 06:30–09:00 h. Second, for each time block the median marker level measured in all studies was calculated, which was then put in the graph.

### 2.2. Bispebjerg Study of Diurnal Variations

#### 2.2.1. Subjects and Study Design

Serum samples from the Bispebjerg study were used for the analysis of cholesterol synthesis and absorption markers. A detailed description of the study design has been published [23]. In summary, twenty-four Caucasian males, with a mean age of 26 years old (range 20–40 years) and mean BMI of 22.9 kg/m^2^ (range 19.6–24.9 kg/m^2^) participated in the study. Subjects spent 24 h at the hospital ward, and standardised meals (low fat, no sugar) were provided at 09:30 h, 13:00 h, and 19:00 h. As both high-fat and high-sugar intake may influence the circadian system [24], it was decided to use meals that were low in fat and sugar to minimize effects on the primary outcomes of the Bispebjerg study. Subjects slept between 23:00 h and 08:00 h in total darkness. Blood samples were collected by cubital venepuncture every three hours from 09:00 h until 09:00 h the next day. The study was executed in accordance with the Helsinki Declaration and written informed consent was signed by all the subjects. Ethical approval was given by the Regional Scientific Ethical Committee of the Capital Region of Denmark (protocol number H-B-2008-011) and the Danish Data Protection agency (journal number 2008-41-1821).

#### 2.2.2. Serum Analyses

Serum concentrations of cholesterol, lathosterol, desmosterol, campesterol, sitosterol, and cholestanol were determined using a gas chromatography flame ionization detector (GC-FID) as described by Mackay et al. [25]. Lathosterol, desmosterol, campesterol, sitosterol, and cholestanol were standardised for cholesterol concentrations (µmol/mmol cholesterol), because they are transported in serum by lipoproteins. The lathosterol/campesterol-ratio at 09:00 h on day 1 was also calculated to classify subjects as cholesterol absorbers (*n =* 8), cholesterol synthesizers (*n =* 8), or intermediate (*n =* 8).

#### 2.2.3. Statistical Analyses

Pearson correlations were calculated to examine associations between the cholesterol absorption and synthesis markers at 09:00 h on day 1. Cosinor analysis for populations was used to examine whether total cholesterol, lathosterol, desmosterol, campesterol, sitosterol, and cholestanol showed a diurnal rhythm [26,27]. A curve was fitted to the data using the following sine and cosine function:

Analyte = M + k1COS(2πt/24) + k2SIN(2πt/24)(1)

Substituting COS(2πt/24) = X and SIN(2πt/24) = Z(2)

gave the final formula: Analyte = M + k1X+ k2Z(3)

Using the GLM procedure in SAS, the model fit was tested and M, k1, and k2 were calculated. Confidence bounds were also calculated and added to the fitted curves. The cosinor model further estimated the mesor, amplitude, and time of peak for total cholesterol and each marker. The mesor represents the rhythm-adjusted mean value of the cosinor curve, and the amplitude represents the difference between the mesor and peak or between the mesor and nadir. It was concluded that a diurnal rhythm was present when the cosinor curve was significant (*cosinor p* < 0.05). In addition, linear mixed models were used to examine whether the marker levels fluctuated significantly over time. Time was defined as fixed factor, subjects as random factor and the best model fit was based on the lowest Akaike Information Criterion. Random intercept models with the identity covariance structures were used. In case of significant time effects (*p* < 0.05), Bonferroni post hoc tests were used to make comparisons between the marker levels at 09:00 h on day 1 and the other time points. The cosinor analyses were conducted in SAS (SAS Institute Inc., Cary, NC, USA) and the other analyses in SPSS version 25 for Mac OS X (SPSS Inc., Chicago, IL, USA).

## 3. Results

### 3.1. Systematic Review

#### 3.1.1. Study Selection

**Figure 1:**
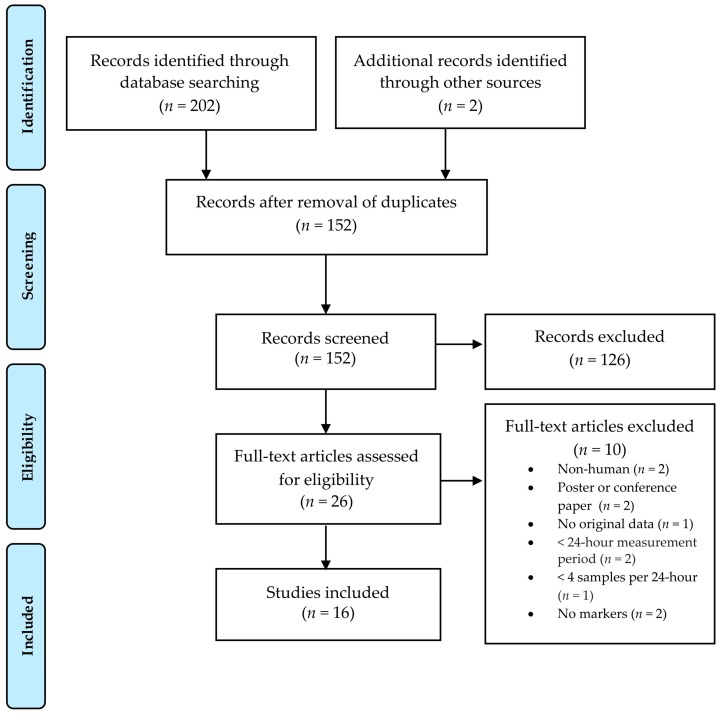
PRISMA flowchart of the study selection process.

Figure 1 provides an overview of the study selection. The search yielded 204 potentially relevant articles and 26 of these were assessed for full-text evaluation. Ultimately, 16 studies met the inclusion criteria and their characteristics are summarized in Table 1.

#### 3.1.2. Lathosterol

Three papers, including five study-arms, have examined the diurnal rhythm of serum lathosterol [40,41,43]. Two studies have examined the diurnal rhythms without treatment [40,43], two studies following cholestyramine (CME) treatment [41,43], and one study following CME plus atorvastatin treatment [43]. See Table S1. 

**Figure 2:**
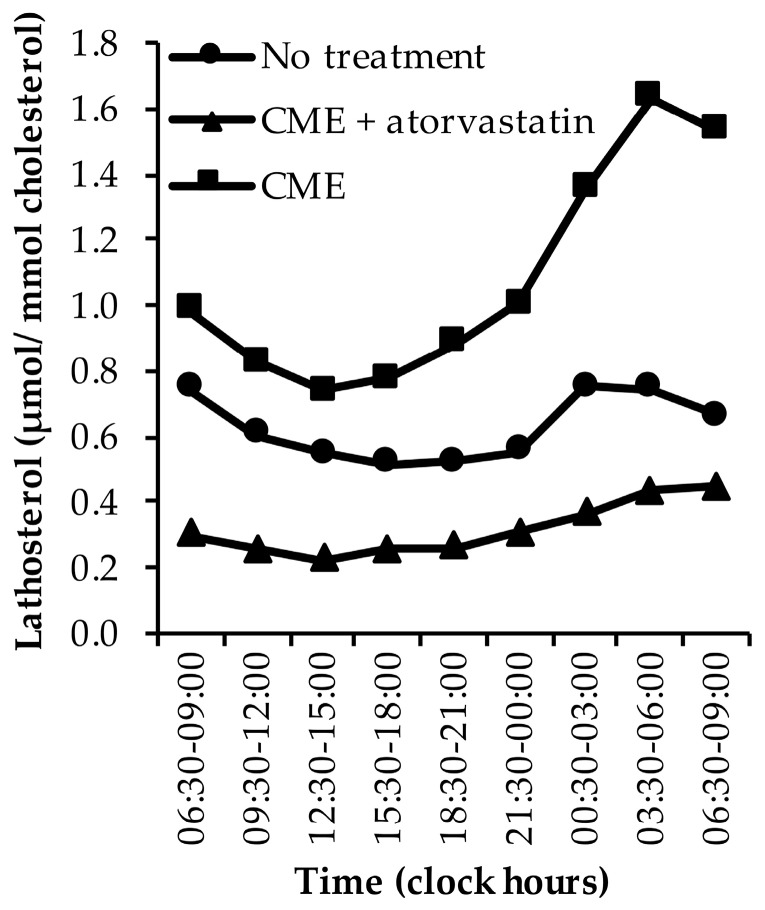
The diurnal rhythm of lathosterol levels in untreated subjects, in subjects treated with cholestyramine (CME) alone, and in subjects treated with CME plus atorvastatin [40,41,43]. Data are presented as medians.

In non-treated subjects, cholesterol-standardised lathosterol levels were lowest during the day and highest during the night (Figure 2) [40,43]. A comparable pattern was observed when subjects received CME with the meals, although cholesterol synthesis rates were increased (Figure 2) [41,43]. In one study, lathosterol levels were almost twice as high on the morning after one-day treatment with CME compared to no treatment [43]. In contrast, lathosterol levels were decreased at all time points following CME plus atorvastatin treatment (Figure 2) [43]. In this study, CME was given with the meals on the first day, and atorvastatin on the morning two days before the study and on the morning of the first and second study day [43]. On the morning of the second day, lathosterol levels were approximately 34% lower compared to no treatment and 66% lower compared to CME alone [43].

#### 3.1.3. Mevalonate (MVA)

Nine papers, including 22 study-arms, have reported on the diurnal rhythm of plasma or serum MVA concentrations [28,30,31,33,34,35,37,38,39]. All studies examined subjects who had not received any treatment [28,30,31,33,34,35,37,38,39], three studies examined the effects of different diets (i.e., high cholesterol intake and fasting) [28,30,31], and three studies examined the effects of statin treatment [37,38,39]. See Table S2. 

**Figure 3:**
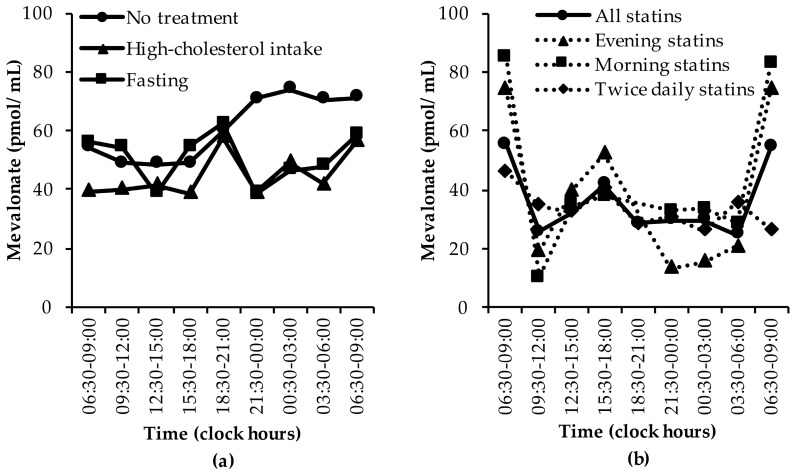
The diurnal rhythm of MVA concentrations: (**a**) in untreated subjects, in subjects who consumed high-cholesterol meals, and in subjects who fasted [28,30,31,33,34,35,37,38,39], and (**b**) in subjects who were treated with statins [37,38,39]. Data are presented as medians.

MVA concentrations were lowest during the day and highest during the night and early morning (Figure 3a). However, two studies found lower MVA concentrations on the morning of the first day compared with the second day [30,35] and another study did not report morning concentrations on the second day [38], which explains the difference in MVA concentrations between the first and second day (Figure 3a).

Consumption of high-cholesterol meals decreased MVA concentrations and the nocturnal peak (Figure 3a) [30,31]. The high-cholesterol graph shows lower MVA morning concentrations on the first than on the second day (Figure 3a), which was caused by differences in the number of observations at each time period due to different sampling times between studies. The high-cholesterol graph also shows increased MVA concentrations in the evening (Figure 3a), which was due to a study that found slightly increased MVA concentrations in the evening when high-cholesterol meals (972 mg cholesterol/day) had been consumed for four weeks [31]. In another study, the 24-h mean MVA concentrations were significantly decreased by 33% when high-cholesterol (1200 mg cholesterol/day) meals had been consumed for three weeks compared to lower-cholesterol meals (550 mg cholesterol/day) [30].

Fasting also decreased MVA concentrations and the nocturnal peak (Figure 3a) [28,30]. In one study, the 24-h mean MVA concentrations were significantly decreased by 55% following a 12-day fast compared to no treatment [30]. One study [28] did not collect samples between 18:30 and 21:00 h and reported lower MVA concentrations than the other study that examined fasting [30], which explains the peak in the fasting graph (Figure 3a). Moreover, fasting MVA concentrations were slightly decreased in the afternoon and at midnight in one study [30], as shown by the nadirs (Figure 3a).

Statin treatment decreased MVA concentrations and the nocturnal MVA peak irrespective of the time of administration (Figure 3b) [37,38,39]. The reason for the high MVA concentrations between 06:30 and 09:00 h in the morning and evening statin group is that only one paper, that found relatively high MVA concentrations compared with other studies (See Table S2), sampled at these time points [37]. In one study, the 24-h mean MVA concentrations were significantly decreased by 33% and 44% following eight-week twice daily lovastatin and simvastatin treatment, respectively, compared to no treatment [38]. Lovastatin decreased the nocturnal MVA peak, which was not present at all after simvastatin treatment, and the ratio between the mean MVA night-time (20:00 h–07:00 h) to daytime (08:00 h–19:00 h) concentration was significantly more reduced by simvastatin (58.3%) than by lovastatin (43.8%) [38]. In another study, the 24-h area under the curve for MVA decreased by approximately 30% and 33% following 14-day morning and evening rosuvastatin treatment, respectively, which was not significantly different [39]. Moreover, the MVA peak concentration observed pre-treatment was reduced by approximately 26% following morning rosuvastatin treatment and by 31% following evening rosuvastatin treatment. These values were not tested for statistical difference [39]. Another study showed that a single pravastatin dose in the morning non-significantly decreased MVA concentrations at 17:00 h, while a single pravastatin dose in the evening borderline significantly decreased MVA concentrations at 23:00 h [37].

#### 3.1.4. Squalene

**Figure 4:**
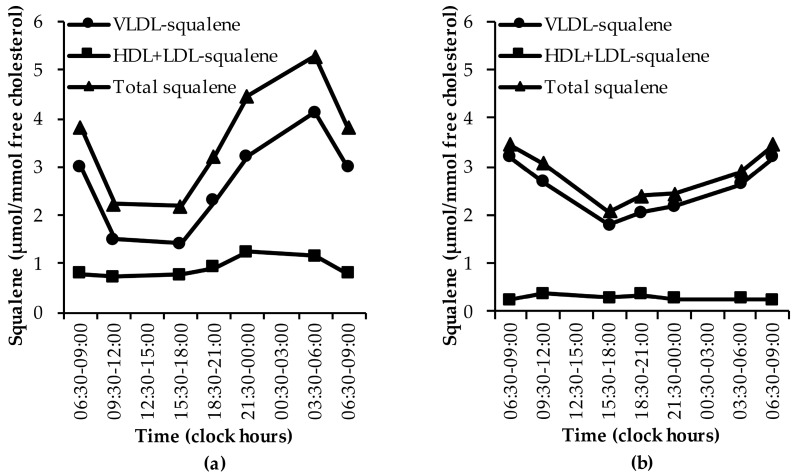
The diurnal rhythm of squalene levels: (**a**) in healthy subjects [29], and (**b**) in subjects with jejunoileal bypass and ileal exclusion [32]. Data are presented as medians.

Two papers were identified that described the diurnal rhythm of squalene. In the first study healthy subjects were investigated, and the other studied subjects with jejunoileal bypass and subjects with ileal exclusion [29,32]. In healthy subjects, squalene levels were lowest during the day and highest during the night (Figure 4a) [29], as shown by the lowest squalene levels in the VLDL and HDL+LDL fractions during the day and highest during the night. This pattern was not observed in subjects with jejunoileal bypass nor in subjects with ileal exclusion (Figure 4b) [32]. See Tables S3 and S4. 

#### 3.1.5. C4

Four papers, including seven study-arms, in which the diurnal rhythm of C4 was evaluated met the inclusion criteria [36,40,42,43]. All papers examined the diurnal rhythm of C4 without any interventions [36,40,42,43]. One study examined cholecystectomized subjects [40], and two other studies examined healthy subjects treated with CME alone and with CME plus atorvastatin [43]. See Table S5. 

**Figure 5:**
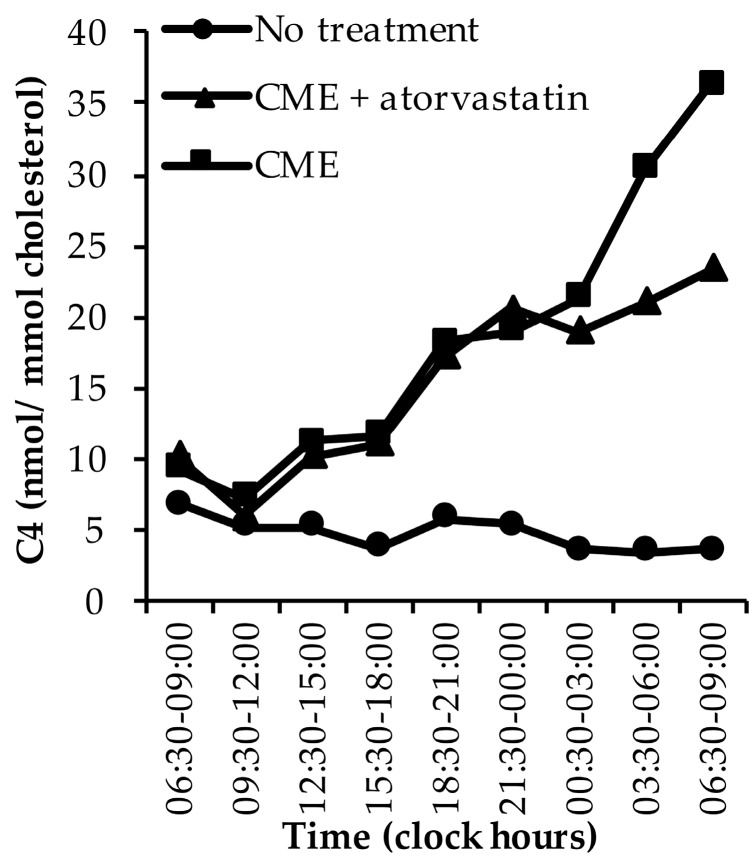
The diurnal rhythm of C4 levels in untreated subjects, in subjects treated with cholestyramine (CME) alone, and in subjects treated with CME plus atorvastatin [40,43]. Data are presented as medians.

In non-treated subjects, C4 levels were highest during the day and evening, and lowest during the night (Figure 5) [40,43]. Peaks were reported at 13:00 h, 21:00 h, 22:00 h, and between 05:30 and 07:00 h [40,43]. One study measured relatively high baseline C4 levels compared to the other time points [43], which explains the difference in morning C4 levels between the first and second day (Figure 5). Two papers only reported absolute C4 concentrations [36,42]. In one paper, C4 concentrations showed a peak around 05:30 h and nadir in the evening [36], while the other paper reported peaks at noon and during the evening and night, and nadirs in the morning and afternoon [42]. In cholecystectomized subjects, cholesterol-standardised C4 levels peaked during the day and evening (around 12:00 h, 22:30 h, and 05:30 h) [40].

CME treatment, given with meals on the first day, ended the rhythm that had been observed without any interventions and increased C4 levels (Figure 5) [43]. This increase had already been observed after the first CME dose in the morning, and levels continued to increase until noon on the second day [43]. One-day CME treatment combined with atorvastatin, which was given on the morning two days before the study and on the morning of the first and second day, also ended the rhythm and increased C4 levels, which was less pronounced compared to CME treatment alone [43]. The increase had already been observed after the first CME plus atorvastatin dose in the morning of the first study day and continued to increase until the afternoon on the second study day (Figure 5) [43]. On the morning of the second day, C4 levels were approximately seven times higher following CME treatment and four times higher following CME plus atorvastatin treatment, compared to no treatment [43].

### 3.2. Bispebjerg Study of Diurnal Variations

Table 2 shows the means ± SD and correlations between the cholesterol-standardised markers at 09:00 h on day 1. Significant negative correlations were found between lathosterol and campesterol, lathosterol and sitosterol, and desmosterol and cholestanol. Significant positive correlations were found between campesterol and sitosterol, campesterol and cholestanol, and sitosterol and cholestanol.

**Figure 6:**
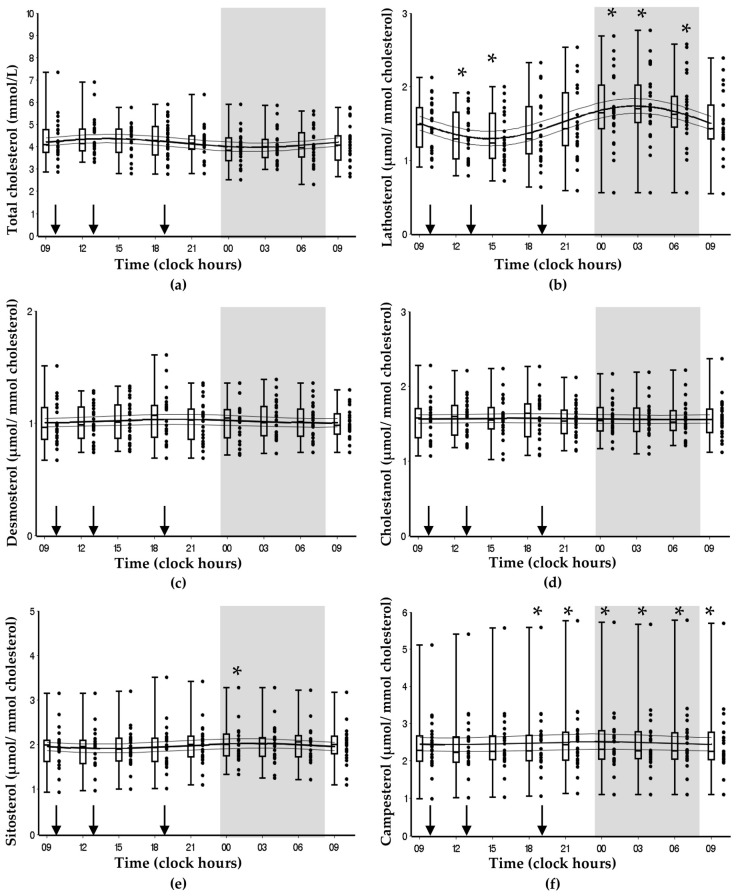
The diurnal rhythms of: (**a**) total cholesterol (*cosinor p* = 0.070), (**b**) lathosterol (*cosinor p* < 0.001), (**c**) desmosterol (*cosinor p* = 0.640), (**d**) cholestanol (*cosinor p* = 0.950), (**e**) sitosterol (*cosinor p* = 0.460), and (**f**) campesterol (*cosinor p* = 0.890) in healthy males (*n* = 24). Data are presented as cosinor curves with confidence bounds. The dots next to the boxplots indicate individual data points. The grey area indicates the sleeping period and the arrows indicate meal timing. * Indicates significant difference (*p* < 0.05) compared with 09:00 h on day 1 using Bonferroni post hoc tests following significant time-effects using a linear mixed model.

The regular day-night rhythms of the subjects were previously confirmed by analysing the 24-h serum melatonin concentrations, which peaked at 03:34 h and were lowest in the afternoon (*cosinor p* < 0.001) [23]. Total cholesterol showed a borderline significant diurnal rhythm (*cosinor p* = 0.070) and significant time-effect (*p* = 0.017), with the highest values during the day and the lowest values during the night (amplitude (amp) = 0.19 mmol/L) (Figure 6a, Table 3). Of the cholesterol synthesis markers, lathosterol has a significant diurnal rhythm (*cosinor p <* 0.001) and a significant time-effect (*p* < 0.001), with a nocturnal peak and nadir in the afternoon (amp = 0.22 µmol/mmol cholesterol) (Figure 6b, Table 3). Desmosterol levels remained relatively stable over time and showed no significant diurnal rhythm (*cosinor p* = 0.640) or significant time-effect (*p* = 0.731) (Figure 6c, Table 3). Of the cholesterol absorption markers, cosinor analysis revealed no significant diurnal rhythms for cholestanol, sitosterol, and campesterol (*cosinor p* > 0.05) (Figure 6d,e,f, Table 3). Moreover, no significant time-effect was found for cholestanol (*p* = 0.896), but significant time-effects were found for sitosterol (*p* < 0.001) and campesterol (*p* < 0.001), which both showed small nocturnal peaks.

The lathosterol/campesterol-ratio at 09:00 h on day 1 was used to classify subjects as cholesterol absorbers (ratio ≤ 0.54), intermediate (0.54 < ratio < 0.73), or cholesterol synthesizers (ratio ≥ 0.73). Overall, the 24-h patterns of lathosterol, desmosterol, cholestanol, sitosterol, and campesterol did not differ between the groups, whereas the mean levels measured at each time point did differ between the groups (data not shown).

## 4. Discussion

Based on a systematic literature search and experimental data, we conclude that plasma markers for endogenous cholesterol synthesis and bile acid synthesis have a diurnal rhythm. No evidence was found for a diurnal rhythm in plasma markers reflecting intestinal cholesterol absorption. The 24-h patterns of the cholesterol synthesis and cholesterol absorption markers were not different for cholesterol synthesizers, cholesterol absorbers, and intermediate subjects.

### 4.1. Endogenous Cholesterol Synthesis

Previous studies have clearly established the diurnal rhythms of plasma MVA, lathosterol, and squalene, which show that cholesterol synthesis is lowest during the day and highest during the night. In the Bispebjerg study, we observed a comparable diurnal rhythm of lathosterol. These findings are in line with studies that used the deuterium incorporation method to examine the diurnal rhythm of the cholesterol fractional synthesis rate (FSR) in healthy people [19,44,45]. These studies reported that cholesterol FSR was lowest during the daytime and highest during the night-time. This agreement in findings also indicates that using non-cholesterol sterol markers to measure cholesterol synthesis is a valid method. Besides MVA plasma concentrations, urinary MVA excretion reflects HMG-CoA reductase activity as well [46]. Interestingly, comparable diurnal patterns have been reported for MVA plasma concentrations and urinary MVA excretion, thereby confirming that both MVA plasma concentrations and urinary MVA excretion reflect endogenous cholesterol synthesis [28,37].

The diurnal rhythm of cholesterol synthesis is mainly regulated by *3*-hydroxy-3-methylglutaryl-CoA (HMG-CoA) reductase, which is the rate-limiting enzyme in the cholesterol synthesis pathway [47]. The diurnal pattern of this enzyme is influenced by circadian clock genes (e.g., *Clock*) and nutrition [47]. Cholesterol synthesis rates were significantly decreased in people who consumed high-cholesterol meals compared to medium- and low-cholesterol meals, indicating a feedback mechanism between increased dietary cholesterol intake and HMG-CoA reductase activity [30,31,48]. The observed decrease in cholesterol synthesis rates following a period of fasting may be attributed to a decreased expression of the sterol regulatory element-binding protein 2 (SREBP2). This is a transcription factor that activates many enzymes in the cholesterol synthesis pathway, including HMG-CoA reductase [49]. Horton et al. has reported a decrease in nuclear forms of SREBP2 in mice liver following a 24-h fast. Consequently, the mRNAs of several genes involved in the cholesterol synthesis pathway were decreased by 40–70% [50]. Under normal circumstances, SREBP2 expression is regulated by the SCAP-INSIG complex. However, when intracellular sterol concentrations are low (e.g., during fasting), this SCAP-INSIG interaction gets disrupted [51]. Previously, it has been shown that SREBP2 expression during fasting is also regulated by sirtuin 1 (SIRT1) [52]. Activation of SIRT1 depends on the presence of NAD^+^, which acts as co-factor in this activation. During a period of fasting, NAD^+^ concentrations are increased and therefore SIRT1 activity is increased as well [53]. In human cells, it was demonstrated that activation of SIRT1 resulted in significantly reduced expression of genes regulated by SREBP2, including HMG-CoA reductase and the LDL-receptor [52]. These findings indicate that SIRT1 reduces SREBP2 expression during a period of fasting and can thereby lead to a decrease in cholesterol synthesis [52].

CME treatment increased lathosterol levels, indicating that HMG-CoA reductase activity was increased by the reduced hepatic cholesterol levels following CME treatment [41,43]. Moreover, no major differences between morning versus evening treatment with rosuvastatin and pravastatin in decreasing 24-h cholesterol synthesis rates were observed, which is likely due to their relatively long half-life in plasma [37,39,54]. The type of statin had a more profound impact on the diurnal rhythm of cholesterol synthesis, as greater reductions in 24-h MVA concentrations have been reported following simvastatin compared to lovastatin treatment [38]. Overall, these findings emphasize that external factors mainly influence the diurnal rhythms of cholesterol synthesis by altering the mean 24-h concentrations and maximum peak value, but not by shifting the period of maximal cholesterol synthesis from night-time to daytime.

Surprisingly, we found no significant diurnal rhythm of desmosterol. The weak baseline correlation between desmosterol and lathosterol combined with the finding that lathosterol and HMG-CoA reductase activity are more strongly correlated than desmosterol and HMG-CoA reductase activity [55], could indicate that lathosterol is the preferred marker to assess cholesterol synthesis. Furthermore, although interventions aimed at reducing intestinal cholesterol absorption have been shown to increase cholesterol synthesis rates, a review has suggested that plant stanol or sterol consumption increased cholesterol-standardised lathosterol levels in 13 out of 17 interventions, while those of desmosterol were only increased in 8 out of 17 interventions [56]. Thereby, in the studies in which lathosterol and desmosterol both significantly increased following plant stanol or sterol consumption, this increase in percentages was often higher in lathosterol than in desmosterol [56]. Perhaps, desmosterol levels remain more stable over time, while lathosterol levels fluctuate more and respond more strongly to interventions. Moreover, desmosterol is formed in the Bloch pathway, whereas lathosterol is formed in the Kandutsch-Russell pathway. Two important enzymes in the regulation of these pathways are 24-dehydrocholesterol reductase (DHCR-24) and 7-dehydrocholesterol reductase (DHCR-7). Of these, DHCR-24 mediates the shift from the Bloch pathway to the Kandutsch-Russell pathway, as well as the conversion of desmosterol to cholesterol, while DHCR-7 mediates the conversion of 7-dehydrodesmosterol to desmosterol, as well as the conversion of 7-dehydrocholesterol to cholesterol [57]. These enzymes are thus involved in both pathways, but the intermediates in both pathways differ. In mice, 7-dehydrocholesterol and 24,25-dihydrolanosterol, both metabolites in the Kandutsch-Russell pathway, had a marked diurnal pattern [58]. Thereby, a diurnal pattern of DHCR-24 was reported in rats fed with CME and with lovastatin [59]. Although Miettinen et al. has suggested that the absolute concentrations of certain free sterols involved in the conversion of lanosterol to cholesterol might fluctuate over a 24-h period [29], more conclusive evidence is needed to confirm whether metabolites in the Kandutsch-Russell and Bloch pathway, as well as DHCR-24 or DHCR-7, are regulated by the circadian system in humans, and could thereby offer an explanation for the different findings for lathosterol and desmosterol.

### 4.2. Intestinal Cholesterol Absorption

Up to now, the influence of the circadian system on human cholesterol absorption has received little attention. In the Bispebjerg study, we found no significant diurnal rhythm of cholestanol, campesterol, and sitosterol levels. Nevertheless, significant time effects were found for campesterol and sitosterol, indicating that their levels did fluctuate over time, but not in a rhythmic 24-h pattern. Animal research has shown that intestinal cells express circadian clock genes, which may control circadian expression of different proteins involved in cholesterol absorption [60]. However, it is currently unknown whether the Niemann-Pick C1-Like 1 protein, essential for intestinal cholesterol absorption [61], or the ATP-binding cassette transporters ABCG5/G8, essential for intestinal cholesterol export [62], are under circadian regulation in humans.

A possible explanation for not finding a diurnal rhythm in cholesterol absorption may be that subjects of the Bispebjerg study consumed low-fat meals. Dietary intake of fat and cholesterol both influence bile acid secretion and cholesterol absorption. For example, high-fat diets increase bile acid excretion, whereas low-fat diets reduce cholesterol absorption in humans [63,64]. Thus, it is possible that other 24-h patterns for cholesterol absorption would have been observed in case the subjects of the Bispebjerg study would have consumed high-fat or high-cholesterol meals.

### 4.3. Bile Acid Synthesis

Based on earlier studies, it is evident that C4 has a diurnal rhythm, with the lowest levels during the night and the highest levels during the day. These findings are partly in agreement with studies that have measured bile acid synthesis as function of CO_2_ output from [26-^14^C]cholesterol, which reported that bile acid synthesis had one peak in the morning and was lowest during the evening and night [20,65]. Possible reasons why only one peak has been reported in these studies could be the small sample sizes (*n =* 3) and relatively complicated method to measure bile acid synthesis. Another study in healthy males reported a mean C4 peak at noon, although C4 also peaked in the morning in some of the subjects [66]. However, this study only collected samples for a 15-h period (07:00–22:00 h). Thus, the nocturnal C4 levels are unknown in these subjects [66].

Previous findings have suggested that the gallbladder has a minimal role in the diurnal regulation of bile acid synthesis, because the rhythm was comparable in cholecystectomized and healthy subjects, and C4 concentrations measured in gallbladder bile were too low to explain the peaks in C4 [40]. Cholesterol 7α-hydroxylase (CYP7A1) is the rate-limiting enzyme in the bile acid synthesis pathway, and can be suppressed by the fibroblast growth factor 19 (FGF19) via the farnesoid X receptor (FXR) [67]. Thus, FGF19 and FXR may be underlying mechanisms regulating the diurnal rhythm of C4. Studies found that postprandial increases in total and unconjugated bile acids were followed by peaks in FGF19, which indicates that FGF19 is secreted in response to the postprandial increases in bile acids [43,68]. Furthermore, comparable rhythms have been reported for FGF19 and C4, although peaks in FGF19 preceded the decline of C4 [68]. A recent study has suggested that the rhythm of C4 can also be regulated via FXR, independent of FGF19 [69]. In this study, subjects received a single dose of a nonsteroidal FXR agonist (Px-102) and the influence of Px-102 on the diurnal rhythms of serum C4, FXR, and FGF19 was investigated. The lowest dose caused C4 levels to decrease by 80% compared with baseline, while FGF19 concentrations only increased when higher Px-102 doses were given. Hence, the decrease in C4 following the lowest dose was not preceded by increased FGF19 levels, which indicates that FXR influenced the diurnal rhythm of C4 independent of FGF19 [69]. In summary, these findings suggest that FXR and FGF19 are both involved in the regulation of the diurnal rhythm of C4, but the exact mechanism remains unclear.

### 4.4. Total Cholesterol

We did not find a significant diurnal rhythm for total cholesterol. Previous studies on the diurnal rhythm of total cholesterol have reported conflicting findings. Some studies did not show a significant diurnal rhythm for total cholesterol concentrations [70,71], whereas other studies did [72,73,74]. However, these latter studies showed little agreement on the timing of peak concentrations, as acrophases were reported in the morning [72], afternoon [73], and at the beginning of the evening [74]. LDL-cholesterol may have a diurnal rhythm with peak concentrations during the day [21,74]. For HDL-cholesterol, several studies have reported that it did not have a significant diurnal rhythm [21,70,71], while other studies did report a significant diurnal rhythm with peaks in the afternoon [73,74].

### 4.5. Discrepancies Between Studies Included in the Systematic Review

Although comparable diurnal patterns have been reported by the studies included in the review, there were some discrepancies with regard to the average marker concentrations and timing of peaks and nadirs. These may be explained by methodological differences between the studies, such as different time intervals between sample collection and analytical methods. For example, one study included in the review did not report a nocturnal MVA peak, which is probably a result of not collecting a nocturnal sample [37]. Thereby, total cholesterol in samples from the Bispebjerg study were previously measured using the colorimetric slide technology [21], while we used the GC-FID. Even though the concentrations and rhythms of total cholesterol were comparable between both analytical methods, there were small differences in the mesor, amplitude, and times of peak and nadir. Therefore, it is expected that the different analytical methods used to measure MVA (e.g., the radio-enzymatic method and gas chromatography-mass spectrometry (GCMS)), lathosterol (e.g., isotope dilution mass spectrometry and GCMS), and C4 (e.g., high-performance liquid chromatography, liquid chromatography-mass spectrometry, and gas chromatography-selected-ion monitoring mass spectrometry) may have resulted in different concentrations, and consequently different mesors, amplitudes and times of peak and nadir between the studies included in the review. Thereby, 14 out of 16 papers measured the diurnal rhythms in less than ten people, which is important considering the large inter-individual variations observed in the diurnal rhythms of cholesterol and bile acid synthesis [40,42,43,66].

The control of external factors might offer an additional explanation for the differences between studies, e.g., meal composition, meal timing, and the number of meals per day differed between the included studies. As dietary fat and cholesterol intake are known to influence cholesterol absorption and cholesterol synthesis, differences in meal composition between the studies may have influenced the rhythms. Furthermore, the impact of meal timing on the diurnal rhythm of cholesterol synthesis has been demonstrated by Cella et al. [44]. In this study, a 6.5-h delay in meal time caused the maximum FSR to delay with six hours after the first day, and with 8.6 h after the third day of delayed meal times [44]. Moreover, a study in which subjects consumed meals with a similar fat and cholesterol content, but either as three or as six meals per day, showed a significantly lower 24-h cholesterol FSR in the subjects who consumed six meals per day [75]. This illustrates that both meal timing and the number of meals per day influenced cholesterol synthesis patterns and rates. In addition to nutrition, information on sleep timing and duration was missing in 12 out of 16 articles. However, sleep timing, sleep duration, and light exposure are known to influence diurnal rhythms, and should preferably be well aligned within and between studies to be able to make good comparisons between findings [2].

### 4.6. Strengths and Limitations

We combined a systematic review with an experimental study, in which serum samples were collected under standardised conditions. This minimized the influence of between-subject variation in exposure to external factors on diurnal rhythms. Nonetheless, the present study does have certain limitations. Given that all serum samples in the experimental study were obtained from young and healthy males, it is unclear to what extent the findings can be extrapolated to other populations, especially since age- and sex-specific differences in the circadian system have been reported [76,77]. Furthermore, the cosinor analysis provided a curve in which the peak and nadir were always twelve hours apart and small fluctuations over time were no longer detectable [23]. The additional mixed model analyses, however, enabled us to examine whether marker levels did fluctuate significantly over the 24-h period, though not in a rhythmic manner. In contrast to most of the studies in the review, the Bispebjerg study had a large sample size, which is relevant considering the inter-individual variation often reported in diurnal studies. In addition, multiple markers reflecting cholesterol synthesis, cholesterol absorption, and bile acid synthesis were examined, thereby providing a complete overview of the influence of the circadian rhythms on cholesterol homeostasis.

## 5. Conclusions

The results of this systematic review show that both cholesterol synthesis and bile acid synthesis have a diurnal rhythm, with a reciprocal pattern. The experimental study confirmed the diurnal rhythm of cholesterol synthesis, which had a nocturnal peak. Although the campesterol and sitosterol levels did fluctuate over time, no diurnal rhythm for cholesterol absorption markers was found under the highly standardised conditions of the Bispebjerg study. This may be due to the low-fat meals consumed by the subjects during the 24-h period. The diurnal rhythms involved in cholesterol homeostasis should be considered when people are exposed to dietary or therapeutic interventions to prevent diseases related to metabolic health. Future research should be carried out to further study the influence of external factors, especially the impact of meal timing and meal composition, on the diurnal rhythms regulating cholesterol homeostasis, as well as whether these rhythms differ for various age groups and health conditions.

## Figures and Tables

**Table 1:** Characteristics of studies included in the systematic review.

First Author (year)	Study Design	Population	Intervention	*n*	Age (years)	Male (%)	Marker	Study Duration (Hours)	Dietary Information				
									Type of Meals	Breakfast	Lunch	Dinner	Other
Kopito (1982) [28]	Experimental, crossover	Healthy subjects	One meal/day No meal	1	40	100	MVA	72	*Day 1 and 2:* standardised dinner *Day 3:* no meal			19:00	
Miettinen (1982) [29]	Longitudinal	Healthy subjects		7	Range: 16–49	N/A	Squalene + methyl sterols	24	Three meals and evening snacks. Fat intake provided approximately 35% of total calories	08:00	12:00	16:00	20:00
Parker (1982) [30]	Experimental, crossover	Subject with hyper-cholesterolemia and ischemic heart disease		1	55	100	MVA	72	Four daily liquid-formula meals in equal portions; caloric intakes adjusted to maintain BW within ± 1.5 kg over 3–5-weeks	08:00	13:00	19:00	10:00
		Subject with hypertri-glyceridemia, obesity, and maturity-onset diabetes	No treatment 12-day fast	1	66	100	MVA	48	*No treatment:* liquid formula diet in five equal portions per day for three weeks *12-day fast:* no dietary intake	08:00	13:00	19:00	10:00/17:00
		Subject with heterozygous familial hyper-cholesterolemia and ischemic heart disease	Moderate cholesterol intake High cholesterol intake	1	45	100	MVA	48	*Moderate intake:* five liquid formula feedings, 550 mg cholesterol/ day, for three weeks *High intake:* five liquid formula feedings, 1200 mg cholesterol/day, for three weeks	08:00	13:00	19:00	10:00/17:00
Parker (1984) [31]	Experimental, crossover	Subject with hypertri-glyceridemia	No treatment Moderate cholesterol intake High cholesterol intake	1	68	100	MVA	72	*No treatment:* eating ad libitum 3 times/day as outpatient *Moderate intake:* five liquid formula feedings, 207 mg cholesterol/day, for four weeks *High intake*: five liquid formula feedings, 972 mg cholesterol/ day, for four weeks	08:00	13:00	19:00	10:00/17:00
Miettinen (1985) [32]	Longitudinal	Subjects with jejunoileal bypass		4	26 ± 8	N/A	Squalene + methyl sterols	24	Low-cholesterol diet, 125 mg cholesterol/ 2400 kcal, 100 g fat/day	08:00	12:00	16:00	20:00
		Subjects with ileal exclusion		4	38 ± 12	N/A							
Scoppola (1991) [33]	Longitudinal	Healthy subjects		1	N/A	N/A	MVA	24	Low fat (<5%), cholesterol-free meals	09:30	12:30	19:00	
Jones (1992) [34]	Longitudinal	Healthy subjects		5	26 ± 4	100	MVA	48	Three self-selected, habitual meals/day for three days prior to and 48 h during the study	08:00 –09:00	12:00 –13:00	18:00 –19:00	
Pappu (1994) [35]	Longitudinal	Healthy subjects		6	30 ± 2	50	MVA	24	Three meals/day (40% fat, 25% CHO, 15% protein)	08:00	12:00	18:00	
		Patients with abetalipoproteinemia		3	24 ± 10	66.6			Three meals/ day (12–15% fat, 70–75% CHO, 13–17% protein)	08:00	12:00	18:00	
Yoshida (1994) [36]	Longitudinal	Patients with cholelithiasis and patients with early cancer of the GI-tract		3	Range: 24 – 28	N/A	C4	24	Normal hospital diets	08:00	12:30	17:30	
Nozaki (1996) [37]	Experimental, crossover	Subjects with heterozygous familial hypercholesterolemia	No treatment Morning pravastatin Evening pravastatin	8	58 ± 9	37.5	MVA	24	Cholesterol intake ± 300 mg/day; 20% fat intake; ratio polyunsaturated to saturated FAs was 1.5; single dose pravastatin (20 mg) taken after breakfast or after dinner	08:00	12:00	18:00	
Pappu (2002) [38]	Experimental, crossover	Subjects with heterozygous familial hypercholesterolemia	No treatment Lovastatin Simvastatin	9 5	41 ± 4 N/A	0	MVA	24	Low-cholesterol, low-fat diet conforming to phase I of the American Heart Association Diet; statins (40 mg) given after breakfast and dinner for eight weeks	08:00	12:00	18:00	
Martin (2002) [39]	Experimental, crossover	Healthy subjects	Morning rovustatin Evening rovustatin	21	N/A	N/A	MVA	24	Individual caloric and fat intake was stabilized; Rosuvastatin (10 mg) taken each morning (~07:00 h) or evening (~18:00 h) for 14 days	N/A	N/A	N/A	
Gälman (2005) [40]	Longitudinal	Healthy subjects (*n* = 5) and cholecystecto-mized subjects (*n* = 3)		8	Range: 25–58	50	C4, latho-sterol	24	Standardised meals	09:00	12:00	18:00	
Persson (2010) [41]	Experimental	Healthy subjects	CME treatment	10	N/A	75	Latho-sterol	33	Standardised meals; CME was taken with meals day 1 (4 × 4 g)	08:30	12:30	16:00	21:30
Steiner (2011) [42]	Longitudinal	Healthy subjects		4	Range: 27–29	50	C4	24	Subjects consumed identical meals	09:15 (day 1) 08:45 (day 2)	11:30	20:15	
Al-Khaifi (2018) [43]	Experimental, crossover	Healthy subjects	No treatment CME CME + atorvastatin	8	Range: 20–45	100	C4, latho-sterol	32	Standardised meals; CME (4 × 4 g) taken before meals day 1; atorvastatin (four daily doses of 40 mg) dose 1 and 2 taken on the morning two days before CME treatment, dose 3 and 4 on the morning of the first and second study day	08:30	13:00	18:00	20:30

*Abbreviations:* BW = body weight; C4 = 7α-hydroxy-4-cholesten-3-one; CHO = carbohydrates; CME = cholestyramine; FA = fatty acids; GI = gastrointestinal; MVA = mevalonate; N/A = data not available.

**Table 2:** Mean ± standard deviation (SD) and correlations between cholesterol-standardised markers reflecting endogenous cholesterol synthesis and intestinal cholesterol absorption at 09:00 h on day 1 in healthy males (*n =* 24).

Marker	Mean ± SD (µmol/mmol cholesterol)	Lathosterol	Desmosterol	Campesterol	Sitosterol
**Lathosterol**	1.5 ± 0.3				
**Desmosterol**	1.0 ± 0.2	0.190			
**Campesterol**	2.4 ± 0.8	−0.521 *	−0.148		
**Sitosterol**	1.9 ± 0.4	−0.438 *	−0.309	0.868 **	
**Cholestanol**	1.6 ± 0.3	−0.368	−0.427 *	0.755 **	0.853 **

* *p* < 0.05; ** *p* < 0.001.

**Table 3:** Results of the cosinor analysis for total cholesterol, and markers reflecting endogenous cholesterol synthesis and intestinal cholesterol absorption in healthy males (*n* = 24).

Marker	Mesor (SE)	Amplitude (SE)	Peak Time (Clock Hours)	Cosinor *p*
**Cholesterol**	4.18 (0.06)	0.19 (0.04)	14:08	0.070
**Lathosterol**	1.52 (0.03)	0.22 (0.02)	02:47	<0.001
**Desmosterol**	1.02 (0.01)	0.02 (0.01)	19:57	0.640
**Campesterol**	2.47 (0.06)	0.04 (0.04)	23:39	0.890
**Sitosterol**	1.97 (0.03)	0.05 (0.02)	01:57	0.460
**Cholestanol**	1.57 (0.02)	0.01 (0.01)	17:00	0.950

*Abbreviations:* SE = standard error. Data presented for cholesterol-standardised marker levels (µmol/mmol cholesterol).

## Supplementary Materials

The following are available online at https://www.mdpi.com/2072-6643/11/7/1439/s1, Table S1: Lathosterol levels (µmol/mmol cholesterol) of the included studies; Table S2: Mevalonate concentrations (pmol/mL) of the included studies; Table S3: VLDL-squalene levels (µmol/mmol free cholesterol) of the included studies; Table S4: LDL+HDL-squalene levels (µmol/mmol free cholesterol) of the included studies; Table S5: C4 levels (nmol/mmol cholesterol) of the included studies.
