# Supplementary material for: Diurnal Variation of Markers for Cholesterol Synthesis, Cholesterol Absorption, and Bile Acid Synthesis: A Systematic Review and the Bispebjerg Study of Diurnal Variations
Source: Nutrients. 2019 Jun 26;11(7):1439. doi: 10.3390/nu11071439 (PMC6683063; doi:10.3390/nu11071439)
Supplement: Supplementary file 1 [file nutrients-11-01439-s001.pdf]

Table S1. Lathosterol levels (µmol/mmol cholesterol) of the included studies.

First author	Intervention	Day 1 (hours)															
		06:30	07:00	07:30	08:00	08:30	09:00	09:30	10:00	10:30	11:00	11:30	12:00	12:30	13:00	13:30	14:00	14:30	15:00
Al-Khaifi	No treatment					0.9			0.7			0.7			0.6			0.6	
[1]	CME					0.9			0.8			0.7			0.6			0.6	
	CME+STAT					0.3			0.3			0.2			0.2			0.2	
Gälman [2]	No treatment						0.6			0.6			0.5			0.5			0.5
Persson [3]	CME					1.1			1.0			0.9			0.8			0.9	

First author	Intervention	Day 1 (hours)															
		15:30	16:00	16:30	17:00	17:30	18:00	18:30	19:00	19:30	20:00	20:30	21:00	21:30	22:00	22:30	23:00	23:30	00:00
Al-Khaifi	No treatment		0.6			0.6			0.5			0.6			0.6			0.6	
[1]	CME		0.6			0.6			0.7			0.8			0.9			1.0	
	CME+STAT		0.3			0.3			0.3			0.3			0.3			0.3	
Gälman [2]	No treatment			0.5			0.5			0.5			0.5			0.6			0.7
Persson [3]	CME		1.1			0.9			1.0			1.1			1.0			1.3	

First author	Intervention	Day 1 (hours)														Day 2	
		00:30	01:00	01:30	02:00	02:30	03:00	03:30	04:00	04:30	05:00	05:30	06:00	06:30	07:00	07:30	08:00	08:30	09:00
Al-Khaifi	No treatment		0.6			0.7			0.7			0.7			0.7			0.7	
[1]	CME		1.1			1.2			1.5			1.3			1.3			1.3	
	CME+STAT		0.3			0.4			0.4			0.4			0.5			0.4	
Gälman [2]	No treatment			0.9			0.8			0.8			0.8			0.7			0.6
Persson [3]	CME		1.5			1.7			1.8			1.8			1.8			1.8	

First author	Intervention	Day 2 (hours)															
		09:30	10:00	10:30	11:00	11:30	12:00	12:30	13:00	13:30	14:00	14:30	15:00	15:30	16:00	16:30	17:00	17:30	18:00
Al-Khaifi	No treatment		0.6			0.6			0.6			0.6			0.7				
[1]	CME		1.3			1.2			1.2			1.4			1.5				
	CME+STAT		0.4			0.4			0.4			0.4			0.4				
Gälman[2]	No treatment			0.5															
Persson[3]	CME		1.7			1.6			1.6			1.7			1.9			1.8	

Values are means. CME = cholestyramine; STAT = statins. N/A = data unknown.

Table S2. Mevalonate concentrations (pmol/mL) of the included studies.

**First**	**Intervention**	**Day 1 (hours)**																		
		**06:00**	**06:30**	**07:00**	**07:30**	**08:00**	**08:30**	**09:00**	**09:30**	**10:00**	**10:30**	**11:00**	**11:30**	**12:00**	**12:30**	**13:00**	**13:30**	**14:00**	**14:30**	**15:00**
Scoppola[4]	No treatment							15.5		12.8				9.5				8.1		
Pappu[5]	No treatment					63.5		50.9		76.6		61.7		61.9		75.2		78.6		65.3
	No treatment[a]					57.0		57.4		57.2		55.6		45.9		55.9		57.9		65.3
Pappu[6]	No treatment					66.3		43.8		43.9		66.7		N/A		N/A		N/A		54.4
	Lovastatin					63.8		45.3		48.0		42.0		67.19		N/A		50.5		N/A
	Simvastatin					47.2		41.5		24.5		27.0		21.1		31.0		15.9		34.2
Parker[7]	No treatment							56.9						91.5						75.1
	No treatment							79.4		68.4		76.3		61.4		57.2		61.2		79.8
	No treatment							45.2		39.0		34.9		37.9		40.1		50.6		94.9
	Fasting							56.1		46.9		61.4		64.3		40.4		36.9		59.9
	HChol meals							62.7		55.3		49.3		27.4		33.6		47.8		44.5
Parker[8]	No treatment					31.0		27.8		11.6		14.3		16.9		18.8		24.9		34.3
	No treatment					29.9		34.9		40.9		37.6		36.1		39.9		35.1		45.3
	HChol meals					39.3		36.3		45.1		35.2		32.4		27.5		38.4		45.3
Jones[9]	No treatment									73.3								48.5		
Kopito[10]	One daily meal																			
	Fasting																			
Nozaki[11]	No treatment					77.8										57.4				
	Morning pravastatin					85.0										57.4				
	Evening pravastatin																			
Martin[12]	Pre-morning treatment	29.7								20.9								25.6		
	Pre-evening treatment	36.2								21.4								26.4		
	Morning rosuvastatin	27.1								10.3								12.2		
	Evening rosuvastatin	19.3								19.0								23.8		

Table S2. *Cont.*

First author	Intervention	Day 1 (hours)																	
		**15:30**	**16:00**	**16:30**	**17:00**	**17:30**	**18:00**	**18:30**	**19:00**	**19:30**	**20:00**	**20:30**	**21:00**	**21:30**	**22:00**	**22:30**	**23:00**	**23:30**	**00:00**
Scoppola[4]	No treatment		6.1				8.1				10.8				10.8				14.2
Pappu[5]	No treatment		52.9		54.1		33.3		53.4		56.3		49.1		57.9		68.7		83.6
	No treatment[a]		59.0		65.8		45.3		66.7		67.8		66.2		68.0		87.2		86.5
Pappu[6]	No treatment		N/A		51.1		34.7		49.2		51.7		75.5		72.0		80.9		70.8
	Lovastatin		N/A		22.5		52.2		34.2		54.1		59.2		55.2		47.2		38.9
	Simvastatin		43.1		40.1		23.0		20.7		23.0		21.0		22.8		20.8		21.7
Parker[7]	No treatment						71.3						63.7						92.6
	No treatment		79.8		93.9		113.4		100.4		107.7		119.9		167.5		151.7		150.0
	No treatment		115.1		98.0		102.4		74.1		77.8		77.2		75.0		76.8		87.9
	Fasting		71.7		44.3		65.1		62.5		68.0		55.1		N/A		61.9		38.6
	HChol meals		41.4		51.5		61.8		69.7		66.5		84.4		N/A		91.0		51.7
Parker[8]	No treatment		40.0		36.5		38.1		37.9		40.0		50.2		63.6		59.7		76.0
	No treatment		56.9		62.3		63.4		70.9		70.9		75.3		83.5		70.4		63.4
	HChol meals		30.2		31.2		36.2		49.3		44.5		38.3		30.2		32.6		39.1
Jones[9]	No treatment						40.3								50.1				
Kopito[10]	One daily meal						10.9								10.3				
	Fasting						13.0								12.9				
Nozaki[11]	No treatment				89.2												84.0		
	Morning pravastatin				55.7												84.1		
	Evening pravastatin				71.4												50.7		
Martin[12]	Pre-morning treatment						35.8								38.5				47.9
	Pre-evening treatment						37.2								35.3				43.5
	Morning rosuvastatin						19.8								26.6				33.0
	Evening rosuvastatin						32.9								11.7				13.4

Table S2. *Cont.*

First author	Intervention	Day 1 (hours)																	
		00:30	01:00	01:30	02:00	02:30	03:00	03:30	04:00	04:30	05:00	05:30	06:00	06:30	07:00	07:30	08:00	08:30	09:00
Scoppola[4]	No treatment				22.9				21.6						18.9				14.2
Pappu[5]	No treatment		129.3		101.8		93.2		84.7		84.5		66.0		94.0		71.0		
	No treatment[a]		87.8		92.1		85.4		101.8		87.4		79.1		81.3		83.6		
Pappu[6]	No treatment		89.1		59.1		81.7		80.9		87.5		56.3		N/A		N/A		
	Lovastatin		40.3		29.7		42.8		31.3		45.0		45.0		N/A		N/A		
	Simvastatin		22.3		22.4		17.5		23.9		33.5		37.9		26.6		N/A		
Parker[7]	No treatment						87.5						48.5						52.2
	No treatment		127.4		166.0		113.2		113.2		108.8		99.6		101.5		78.7		
	No treatment		147.1		196.0		129.8		117.3		95.2		104.2		94.5		79.6		79.2
	Fasting		56.1		47.8		45.4		57.9		80.3		56.4		55.3		61.9		
	HChol meals		63.2		61.8		71.3		65.8		57.9		49.6		55.7		56.8		73.5
Parker[8]	No treatment		68.2		62.6		62.4		56.3		55.3		45.2		N/A		N/A		
	No treatment		53.7		46.0		44.0		40.5		39.4		37.3		35.8		35.8		
	HChol meals		35.8		35.1		36.9		34.0		29.8		32.0		27.9		N/A		
Jones[9]	No treatment				66.6								68.4						
Kopito[10]	One daily meal				34.4								54.2						
	Fasting				10.6								8.1						
Nozaki[11]	No treatment																76.5		
	Morning pravastatin																82.4		
	Evening pravastatin																74.1		
Martin[12]	Pre-morning treatment				43.2								25.0						
	Pre-evening treatment				47.5								31.3						
	Morning rosuvastatin				33.4								30.1						
	Evening rosuvastatin				15.5								21.0						

Table S2. *Cont.*

First author	Intervention	Day 2 (hours)																	
		09:30	10:00	10:30	11:00	11:30	12:00	12:30	13:00	13:30	14:00	14:30	15:00	15:30	16:00	16:30	17:00	17:30	18:00
Parker[7]	No treatment						47.9						48.7						57.8
Jones[9]	No treatment		57.6								46.7								45.8
Kopito[10]	One daily meal		39.7								18.0								15.1
	Fasting		7.6								7.9								N/A
Nozaki[11]	No treatment																		
	Morning pravastatin																		
	Evening pravastatin								55.7								75.9		

First author	Intervention	Day 2 (hours)																	
		18:30	19:00	19:30	20:00	20:30	21:00	21:30	22:00	22:30	23:00	23:30	00:00	00:30	01:00	01:30	02:00	02:30	03:00
Parker[7]	No treatment						58.4						62.1						125.1
Jones[9]	No treatment								51.2								65.0		
Kopito[10]	One daily meal								12.2								32.2		

First author	Intervention	Day 2 (hours)																	
		03:30	04:00	04:30	05:00	05:30	06:00	06:30	07:00	07:30	08:00	08:30	09:00	09:30	10:00	10:30	11:00	11:30	12:00
Parker[7]	No treatment						56.5						65.7						87.8
Jones[9]	No treatment						58.4												
Kopito[10]	One daily meal						46.3								32.8				

First author	Intervention	Day 3 (hours)																	
		12:30	13:00	13:30	14:00	14:30	15:00	15:30	16:00	16:30	17:00	17:30	18:00	18:30	19:00	19:30	20:00	20:30	21:00
Parker[7]	No treatment						81.9						71.2						90.0
Kopito[10]	One daily meal				23.3														

First author	Intervention	Day 3 (hours)																	
		21:30	22:00	22:30	23:00	23:30	00:00	00:30	01:00	01:30	02:00	02:30	03:00	03:30	04:00	04:30	05:00	05:30	06:00
Parker[7]	No treatment						123.1						45.3						35.0

Values are means. HChol = high cholesterol. N/A = data unkown. [a] Subjects with phenotypic abetalipoproteineamia.

Tabel S3. VLDL-squalene levels (µmol/mmol free cholesterol) of the included studies.

First author	Subgroup	Day 1 (hours)																	
		06:30	07:00	07:30	08:00	08:30	09:00	09:30	10:00	10:30	11:00	11:30	12:00	12:30	13:00	13:30	14:00	14:30	15:00
Mietinnen [13]	Healthy subjects				3.0								1.5						
Mietinnen [14]	Jejunoileal bypass				4.7								3.8						
	Ileal exclusion				1.7								1.6						

First author	Subgroup	Day 1 (hours)																	
		15:30	16:00	16:30	17:00	17:30	18:00	18:30	19:00	19:30	20:00	20:30	21:00	21:30	22:00	22:30	23:00	23:30	00:00
Mietinnen [13]	Healthy subjects		1.4								2.3								3.2
Mietinnen [14]	Jejunoileal bypass		2.1								2.1								2.6
	Ileal exclusion		1.5								1.9								1.7

First author	Subgroup	Day 1 (hours)																	
		00:30	01:00	01:30	02:00	02:30	03:00	03:30	04:00	04:30	05:00	05:30	06:00	06:30	07:00	07:30	08:00	08:30	09:00
Mietinnen [13]	Healthy subjects								4.1										
Mietinnen [14]	Jejunoileal bypass								3.5										
	Ileal exclusion								1.8										

Values are means.

Tabel S4. LDL+HDL-squalene levels (µmol/mmol free cholesterol) of the included studies.

First author	Subgroup	Day 1 (hours)																	
		**06:30**	**07:00**	**07:30**	**08:00**	**08:30**	**09:00**	**09:30**	**10:00**	**10:30**	**11:00**	**11:30**	**12:00**	**12:30**	**13:00**	**13:30**	**14:00**	**14:30**	**15:00**
Mietinnen [13]	Healthy subjects				0.8								0.7						
Mietinnen [14]	Jejunoileal bypass				0.3								0.5						
	Ileal exclusion				0.1								0.3						

First author	Subgroup	Day 1 (hours)																	
		**15:30**	**16:00**	**16:30**	**17:00**	**17:30**	**18:00**	**18:30**	**19:00**	**19:30**	**20:00**	**20:30**	**21:00**	**21:30**	**22:00**	**22:30**	**23:00**	**23:30**	**00:00**
Mietinnen [13]	Healthy subjects		0.8								0.9								1.2
Mietinnen [14]	Jejunoileal bypass		0.4								0.4								0.3
	Ileal exclusion		0.2								0.2								0.2

First author	Subgroup	Day 1 (hours)																	
		**00:30**	**01:00**	**01:30**	**02:00**	**02:30**	**03:00**	**03:30**	**04:00**	**04:30**	**05:00**	**05:30**	**06:00**	**06:30**	**07:00**	**07:30**	**08:00**	**08:30**	**09:00**
Mietinnen [13]	Healthy subjects								1.2										
Mietinnen [14]	Jejunoileal bypass								0.3										
	Ileal exclusion								0.2										

Values are means.

Tabel S5. C4 levels (nmol/mmol cholesterol) of the included studies.

First author	Intervention	Day 1 (hours)																	
		06:30	07:00	07:30	08:00	08:30	09:00	09:30	10:00	10:30	11:00	11:30	12:00	12:30	13:00	13:30	14:00	14:30	15:00
Al-Khaifi [1]	No treatment					11.0			7.2			5.8			6.7			5.6	
	CME					9.4			5.9			8.6			10.4			12.2	
	CME + STAT					10.2			5.4			6.5			9.0			11.3	
Gälman [2]	No treatment						2.6			2.2			4.6			4.9			3.6
	No treatment[a]						3.4			2.7			8.3			5.3			4.5

First author	Intervention	Day 1 (hours)																	
		15:30	16:00	16:30	17:00	17:30	18:00	18:30	19:00	19:30	20:00	20:30	21:00	21:30	22:00	22:30	23:00	23:30	00:00
Al-Khaifi [1]	No treatment		4.8			5.9			6.4			6.1			7.2			5.3	
	CME		11.7			11.8			16.2			20.2			19.1			18.9	
	CME + STAT		10.3			11.9			15.1			19.4			19.0			22.0	
Gälman [2]	No treatment			2.8			2.4			3.8			5.6			5.4			3.5
	No treatment[a]			3.8			3.2			3.2			5.1			5.7			4.5

First author	Intervention	Day 1 (hours)																Day 2	
		00:30	01:00	01:30	02:00	02:30	03:00	03:30	04:00	04:30	05:00	05:30	06:00	06:30	07:00	07:30	08:00	08:30	09:00
Al-Khaifi [1]	No treatment		5.2			4.4			4.7			7.0			6.5			5.1	
	CME		19.7			23.0			24.2			36.5			37.6			35.1	
	CME + STAT		20.5			17.3			19.7			22.3			24.4			22.3	
Gälman [2]	No treatment			2.8			2.7			2.2			1.9			2.2			2.1
	No treatment[a]			5.3			4.6			7.4			5.2			5.0			3.2

First author	Intervention	Day 2 (hours)																	
		09:30	10:00	10:30	11:00	11:30	12:00	12:30	13:00	13:30	14:00	14:30	15:00	15:30	16:00	16:30	17:00	17:30	18:00
Al-Khaifi [1]	No treatment		3.9			5.6			6.8			6.2			4.2				
	CME		38.7			49.3			46.6			41.3			29.3				49.3
	CME + STAT		20.7			24.5			27.8			25.7			20.3				24.5
Gälman [2]	No treatment			1.8															
	No treatment[a]			2.6															

Values are means. CME = cholestyramine; STAT = statins. [a] Cholecystectomized subjects.
